# Musical Creativity “Revealed” in Brain Structure: Interplay between Motor, Default Mode, and Limbic Networks

**DOI:** 10.1038/srep20482

**Published:** 2016-02-18

**Authors:** David M. Bashwiner, Christopher J. Wertz, Ranee A. Flores, Rex E. Jung

**Affiliations:** 1 University of New Mexico, Department of Music, Albuquerque, NM, 87102, United States; 2 University of New Mexico, Department of Neurosurgery, Albuquerque, NM, 87102, United States; 3 University of New Mexico, Department of Psychology, Albuquerque, NM, 87102, United States

## Abstract

Creative behaviors are among the most complex that humans engage in, involving not only highly intricate, domain-specific knowledge and skill, but also domain-general processing styles and the affective drive to create. This study presents structural imaging data indicating that musically creative people (as indicated by self-report) have greater cortical surface area or volume in a) regions associated with domain-specific higher-cognitive motor activity and sound processing (dorsal premotor cortex, supplementary and pre-supplementary motor areas, and planum temporale), b) domain-general creative-ideation regions associated with the default mode network (dorsomedial prefrontal cortex, middle temporal gyrus, and temporal pole), and c) emotion-related regions (orbitofrontal cortex, temporal pole, and amygdala). These findings suggest that domain-specific musical expertise, default-mode cognitive processing style, and intensity of emotional experience might all coordinate to motivate and facilitate the drive to create music.

Creative behaviors are often treated as mysterious—musically creative behaviors perhaps especially. For example, the entire repertoire of Gregorian Chant is reputed to have been sung to Pope Gregory by a dove, while the Devil’s Trill Sonata is said to have come to Tartini in a dream, played by the Devil himself. Creative “revelations” of this sort—often called Big C creativity^1^—are no doubt difficult to study scientifically. But everyday creative behaviors—little c—are arguably within reach.

Progress has been made in recent years toward understanding little c creative behavior from the neuroscientific perspective. By definition, “creativity” has been understood to refer to the production of things and ideas that are both novel and useful^1^. Multiple subprocesses are believed to be involved in creative mentation, including the ability to both focus and defocus the attention^2^, to generate variations and select between them^3^, to regress to primary-process types of consciousness^4^, to search memory stores either deliberately or spontaneously^5^, and to do so using either cognitive or emotional search processes^6^.

One brain network that has been proposed to be especially central to creative functioning is the default mode network (DMN)^7^,^8^. The DMN is composed of regions such as the dorsomedial prefrontal cortex (dMPFC), ventromedial prefrontal cortex (vMPFC), lateral temporal cortex (LTC), posterior cingulate, and inferior parietal lobule (IPL)—regions which, when a subject is not given an explicit task, tend to increase in activation relative to baseline^9^. The regions of this network also tend to be implicated in a number of cognitive capacities related to creativity, such as divergent thinking^7^,^8^, self-referential thinking^10^, affective reasoning^6^, mind wandering^11^, and mental simulation^12^. It might be expected, therefore, that creative behavior of a musical nature would also implicate the DMN.

The functional imaging literature has indeed implicated the DMN in musically creative behavior—at least improvisation^13^ (which, because it is instantaneous, is more easily studied in the scanner than are drawn-out processes like orchestral scoring and songwriting). Limb and Braun^14^, for instance, had professional jazz pianists improvise while in the scanner, finding that improvisation (compared with exact replication of a melody or scale) correlated with enhanced activity in medial prefrontal regions (MPFC) and diminished activity in lateral prefrontal regions (LPFC). In a related study from the same laboratory, Liu *et al.*^15^ compared improvised vs. memorized rap performances by professional freestyle artists, again finding significant activation in the MPFC, which was in turn negatively correlated with activity in dorsolateral prefrontal cortex (dLPFC). Supporting these findings, Pinho *et al.*^16^ studied trained pianists with more vs. less experience improvising (compared to playing classically), finding that, during an improvisation, experienced improvisers showed reduced activity in right-hemisphere regions implicated in top-down cognitive control, such as the dLPFC and inferior frontal gyrus (IFG). At the same time, these musicians showed increased functional connectivity in numerous prefrontal, premotor, and motor regions.

These findings suggest that, while regions in the DMN are frequently implicated in studies of musical improvisation and creativity, certain other regions outside of the DMN are also implicated, notably dorsal premotor cortical regions (dPMC) and the supplementary and pre-supplementary motor areas (SMA and pre-SMA). Indeed, all imaging studies of musical improvisation to date implicate at least one of these regions^13^,^14^,^15^,^16^,^17^,^18^,^19^,^20^,^21^. These regions are highly interconnected with one another, both anatomically^22^ and functionally^16^,^23^. Furthermore, they connect to one another across the hemispheres by means of a portion of the corpus callosum (CC) that has been demonstrated to be larger in musicians compared to nonmusicians^24^. The dPMC, SMA, and pre-SMA are all implicated in higher-cognitive aspects of motor control^25^, particularly as they extend more rostrally within the frontal lobe^22^. Thus while the DMN appears to be one system frequently recruited in musical improvisation tasks, regions outside of this network might also be expected to be involved.

Finally, because music and emotion are so intertwined^26^,^27^, it would not be surprising if musically creative people were more emotionally sensitive to music than controls. While Ulrich *et al.*^28^ found reduced activation in the amygdala and MPFC when subjects entered flow states, those flow states were induced by performing mathematical calculations rather than creating music. We hypothesized that we might see brain-behavior associations with musical creativity in limbic and paralimbic regions indexing not the *capacity* to create, but the *drive* to do so.

To our knowledge, no previous studies have examined brain structure as it relates to specifically *creative* musical behavior—although numerous studies have examined brain structure as it relates to musical experience more generally. The planum temporale (PT) is larger on the left side of the brain than on the right in humans generally, and this appears to be more the case in musicians compared to nonmusicians^29^. The CC tends to be thicker in musicians who begin their training at an early age, including in regions of the CC that connect the motor and premotor cortices across the hemispheres^24^. Likewise the arcuate fasciculus, which connects the posterior temporal lobe region to the premotor region of the frontal lobe, has been shown to be thicker and more structurally sound in musicians compared to nonmusicians^30^. Finally, cortical gray matter has been found to be thicker in various regions of the brains of trained musicians, including primary auditory and motor regions and numerous prefrontal cortical regions^31^.

The present study reports on the structural correlates of self-reported musical creativity in a sample of 239 subjects with expertise in the STEM fields (science, technology, engineering, and mathematics). On the assumption that regions shown to be functionally active during musically creative tasks are candidates for structural enhancement, we hypothesized that subjects reporting high levels of musical creativity would show greater surface area in regions affiliated with the DMN (such as dMPFC and LTC), higher-cognitive motor regions (such as dPMC, SMA, and pre-SMA), and limbic and paralimbic regions (such as amygdala and OFC).

## Results

Any deviations from the initial sample were due to missing behavioral data and were excluded before analysis was conducted. While the “Musical Creativity Questionnaire” we designed for this study collected data on many aspects of subjects’ past musical experiences (see Supplementary Fig. S1 online), the number of subjects in our pool with experience playing music and being musically creative was relatively small (N = 113 of 239), and we therefore chose to restrict our study of the data to one very general question about the subjects’ self-rated degree of musical creativity (Table 1; see Methods section for discussion).

Our measure of musical creativity was weakly but significantly correlated with other measures of creativity such as the Creative Achievement Questionnaire (r = 0.28, P = 0.001) and the personality trait Big Five Aspects Scale Openness-Intellect (r = 0.19, P = 0.0103; Table 2), both of which have been shown to be correlated with behavioral creativity measures^32^.

**Figure 1:**
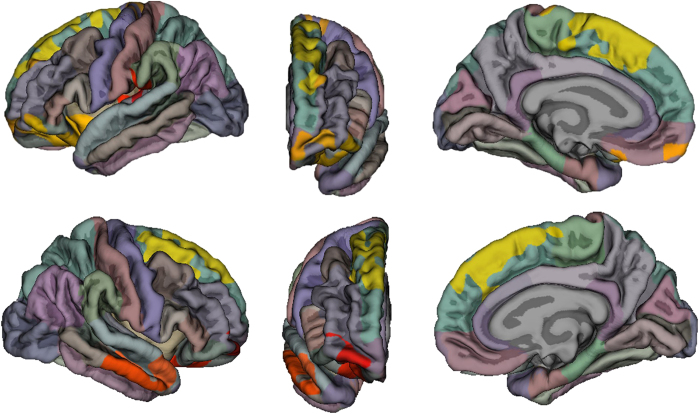
Regions in which surface area correlated significantly (yellow, orange, red) with musical creativity ratings across the entire sample (N = 239).

In both hemispheres, we found significant clusters of greater cortical surface area at P < 0.05, corrected for multiple comparisons, that had a positive correlation with higher musical creativity ratings (Fig. 1 and Table 3). These include bilateral dorsomedial superior frontal gyrus (SFG) (P = 0.00010), bilateral OFC (left, P = 0.00010; right P = 0.01900), left planum temporale region (PT) (P = 0.03840), and right middle temporal gyrus (MTG) (P = 0.00510). Musical creativity ratings were also found to correlate significantly with subcortical volume in left amygdala (F = 3.4, p = 0.02, Beta = 0.17).

## Discussion

Our results indicate that several brain regions show increased surface area in subjects reporting high levels of musical creativity (i.e., high levels of having “improvised or written original music”). These regions include (a) bilateral dorsomedial SFG, extending from the dPMC and SMA posteriorly (BA 6) to well anterior of the rostral portions of these regions in BAs 8 and 9; (b) bilateral OFC, with greater representation in the left hemisphere, extending to the medial wall of the frontal pole (in the left hemisphere) as well as posteriorly as far back as anterior insula (in both hemispheres); (c) right MTG, extending into the superior temporal gyrus (STG) at the pole; and (d) left planum temporale region (PT). We also examined subcortical volume, finding increased volume in (e) left amygdala.

A main finding of this study is that high creativity correlated with enhanced surface area in three out of four nodes of the dMPFC subsystem of the DMN^9^,^33^—namely, dMPFC, LTC, and temporal pole (TP). The DMN has been frequently implicated in studies of creativity generally^7^,^8^, as well as in studies specifically focused on musical creativity^14^,^15^,^16^. The dMPFC subsystem of the DMN in particular has been implicated in reflecting on one’s own internal state and that of others^10^,^33^, making aesthetic judgments^3^,^34^, and emotional reasoning^6^. Thus our findings suggest that this subsystem of the DMN may be integral to musical creativity—more precisely that musico-creative experiences may either lead to, or result from, enhanced brain surface area in the DMN’s dMPFC-subsystem.

Though medial within the prefrontal cortex, the SMA and pre-SMA, along with the dPMC on the dorsal surface, are not frequently included in the DMN^9^. Instead, these regions tend to be implicated in active tasks, particularly tasks related to motor performance and event sequencing. As noted, all imaging studies on the subject of musical improvisation of which we are aware have implicated at least one of these three regions^13^,^14^,^15^,^16^,^17^,^18^,^19^,^20^,^21^, and studies of music perception and production more generally implicate these regions, particularly for tasks related to rhythmic perception^25^,^35^, rhythmic motor imagery^36^, and rhythmic motor production^37^,^38^. These regions have been reported to be implicated in higher-cognitive aspects of motor sequencing, particularly so in their more rostral extents^22^. The pre-SMA has been linked to the perception^35^ and production^37^,^38^ of greater complexity in music; it has also been implicated in freely chosen motor activities, particularly when timing is an issue^17^,^39^. The SMA proper is less implicated in studies of musical production than in musical perception; it has been proposed, however, to be involved in executing the movements that are planned in the pre-SMA^39^, and Narayana *et al.*^23^ report that it coactivates with MTG and the transverse temporal region (including PT) specifically for cognitive aspects of motor tasks. The dPMC is implicated in most music perception and production tasks, and has been proposed to be involved in “extracting higher-order features of the auditory stimulus … in order to implement temporally organized actions”[25, p.554]. Thus all three regions are implicated in higher-cognitive motor processing, and collectively they may represent enhancements less general to creativity and more specific to musical creativity.

Another region showing increased surface area and implying plasticity of a domain-specific nature is the left PT region. This region has been called a “computational hub”^40^ and is believed to perform complex computations upon sounds, translating spectrotemporal sonic information into inferences about objects and their locations in space^25^,^40^. The PT is highly asymmetrical in humans, with larger surface area in the left hemisphere, especially in musicians^29^—who additionally show enhanced processing of speech sounds that occur at extremely rapid rates (up to 40 Hz)^41^. Trained musicians tend to use the left PT region more than nonmusicians do when listening to music^42^, suggesting to Meyer and colleagues that “highly proficient musicians scan the incoming acoustic signal with higher temporal resolution in order to process the music in a more fine-grained mode”[41, p.118]. Chen *et al.*^38^ further report enhanced functional connectivity between the PT and the dPMC, on the left in particular, for trained musicians. Taken together, these findings can be interpreted to indicate enhanced coordination of sound processing in the temporal lobe with higher-cognitive motor sequencing in the frontal lobe in the brains of musically creative individuals.

The MTG and TP also showed enhanced surface area in musically creative individuals. Both regions are frequently implicated in the default mode network, particularly the dMPFC subsystem^9^,^33^, and Wei *et al.*^43^ report that resting state functional connectivity between MTG and MPFC is higher among more creative individuals. The MTG has been implicated in the perception of the semantic content of music^44^, and the temporal pole has been implicated in the experience of emotion in music^45^,^46^. Both regions have been found to be more responsive to musico-structural violations in musicians compared to nonmusicians^42^,^47^,^48^. Brown *et al.*^19^ report that the temporal pole is active when singers harmonize spontaneously with another voice—suggesting to the authors that the superior part of the temporal pole may be a type of “tertiary auditory cortex specialized for higher-level pitch processing related to complex melodies and harmonies, including the affective responses that accompany such processing” (p.371). In sum, both MTG and TP are implicated in both default-mode processing and in music perception, particularly semantic and affective types of music perception. The enhanced surface area seen in these regions in musically creative individuals, therefore, may represent a neural link between default-mode processing, music perception, and emotion.

The OFC is another region in which we saw enhanced surface area bilaterally, and which has been frequently implicated in emotional responses to music^45^,^49^,^50^. Damage in this region has been reported to impair creativity^51^, and our laboratory reports elsewhere^52^ that cortical thickness in left OFC correlates with enhanced divergent thinking and openness. As explained by Kringelbach^53^, the OFC is particularly implicated in integrating sensory input with reward value, and Bechara *et al.*^54^ have demonstrated that the OFC is integral to incorporating emotional and somatosensory input into decision-making processes. Brown *et al.*^45^ note that the OFC, TP, and amygdala are all highly interconnected, suggesting a role for their involvement in musico-affective experience. For our subjects, enhanced surface area in the OFC may therefore be interpreted to indicate enhanced emotional engagement with music—perhaps undergirding the drive to create.

Further support for this interpretation comes from our finding of increased left-hemisphere amygdala volume correlating with musical creativity. The amygdala is perhaps the most frequently implicated brain structure in studies of musical emotion, correlating with emotional responses related to fear, joy, pleasure/displeasure, sadness, tension, and unexpectedness. Liu *et al.*^15^ report enhanced functional connectivity between pre-SMA and left amygdala during improvisation, and further enhanced connectivity between left amygdala and numerous other regions involved in music perception and execution, such as insula, IFG, IPL, and anterior cingulate. In a study by Salimpoor *et al.*^50^, the amygdala and OFC also showed increased functional connectivity with the nucleus accumbens—the brain’s “reward center”—correlated with the degree to which subjects liked pieces of music. Collectively, these results situate the amygdala within a “hedonic evaluation network”—in which music is perceived and parsed in the STG and PT, is engaged with at higher levels via dPMC, SMA, and pre-SMA, and finally is evaluated via the coordination and enhanced functional connectivity of OFC, TP, and amygdala.

There are several limitations to the conclusions drawn here. First, though we examined only brain structure, we interpreted our findings based in part upon the functional imaging literature. It remains uncertain to what extent function and structure in the brain are correlated. Second, we examined surface area of the brain rather than volume or thickness. Surface area can index the size either of intracortical elements or of local subcortical factors^55^, and thus should be interpreted with caution. Nevertheless, surface area is the most stable of the three measures across time^55^, making it potentially the most valid for a cohort of subjects spanning almost two decades of age difference. Third, because our subjects were all young adults, these results might not generalize to children and older adults. Nevertheless, we chose to examine subjects at a point in time when their brains were for the most part fully formed but had not yet begun to demonstrate the structural effects of aging. Fourth, our subjects had (for other reasons) been selected for expertise in the STEM fields, and it would be important to replicate this study using subjects drawn from fields more associated with the arts and humanities. Fifth, this study is correlational and not causal, and it is therefore not possible to determine whether the brain morphometry patterns found for more musically creative individuals led them to create more, or whether creating more led to the brain morphometry patterns seen here. All that can be deduced is that the patterns found correlate with enhanced musical creativity (as indicated by self-report). Sixth, the method used for assessing musical creativity relied entirely on self-report, which is always of questionable reliability, although the correlation of this measure with other well-validated measures of creativity—namely the Creative Achievement Questionnaire and the Big Five Aspects Scale for Openness-Intellect—increases our confidence in its construct validity. Seventh, we were not able to distinguish between different types of musical creativity, such as improvisation vs. orchestral composition vs. songwriting—which may involve very different sets of cognitive processes, and hence very different neural processes. Finally, the current study lacked explicit test-retest reliability measures, which is a weakness of our approach. We are currently undertaking such studies and anticipate reporting on these in future research reports.

By way of outro, it may be of value to reflect upon the creative process as described by composer Johannes Brahms. Brahms stated that, when truly inspired, a “finished product” would often be “revealed” to him “measure by measure.” Notably, he had to be “in a semi-trance condition to get such results—a condition when the conscious mind is in temporary abeyance and the subconscious is in control, for it is through the subconscious mind…that the inspiration comes”^56^. However anachronistically, we may interpret this description as referring to what we now call the default mode of brain activity. Nevertheless, we should not assume this to be the entirety of musical creativity, for, as Brahms pointed out, “a composer must have mastered the technic [*sic*] of composition, form, theory, harmony, counterpoint, instrumentation”[56, p.6]. He insisted, “my compositions are not the fruits of inspiration alone, but of severe, laborious and painstaking toil”[56, p.59].

Creative behaviors—of both big C and little c types—are among the most complex that humans engage in. They involve not only domain-general capacities, such as the ability to defocus the attention and let ideas “reveal” themselves into consciousness seemingly of their own accord, but also highly intricate, domain-specific knowledge and skill—developed over years of practice—all motivated by the affective drive to create. This study highlights structural imaging data indicating that self-reported experience being musically creative correlates with greater cortical surface area or volume in (a) domain-general creative-ideation regions organized around the default mode network (dMPFC, MTG, TP), (b) domain-specific regions frequently recruited for musical tasks (dPMC, SMA, pre-SMA, PT), and (c) emotion-affiliated regions (OFC, TP, and amygdala). These findings suggest that default-mode cognitive processing style, domain-specific musical expertise, and intensity of emotional experience are likely coordinated to both facilitate and motivate the drive to create music.

## Methods

This study was conducted in accordance with the principles in the Declaration of Helsinki. The study was approved by the Institutional Review Board of the University of New Mexico (IRB 11-531). All subjects provided written informed consent before collection of any data and subsequent data analysis.

### Subjects

Two hundred and thirty-nine subjects working or studying in the STEM fields were recruited for the present study. Subjects ranged from 16 to 32 years of age (21.9 +/− 3.5 years) and were well-matched by gender (123 males, 116 females). They were recruited through postings in departments and classrooms at the University of New Mexico, at local high schools, and at various STEM-related places of business. Prior to entry into the study, subjects were screened by a questionnaire and met no criteria for neurological or psychological disorders that would impact experimental hypotheses (e.g., learning disorders, traumatic brain injury, major depressive disorder). Subjects were also screened for conditions that would prohibit undergoing an MRI scan (e.g., metal implant, orthodontic braces, claustrophobia). Subjects were compensated 100 dollars for their participation in the study.

### Behavioral Measures

Subjects were administered a musical creativity questionnaire consisting of four sections inquiring about different aspects of their musical background (see Supplementary Fig. S1 online). The first set of questions asked whether the subject had ever practiced a musical instrument daily or several hours per day, and, if so, which instruments were practiced, for how many years, for how many hours per day, whether such study was formal or informal, and whether the dominant mode of learning was through written notation or by ear. A second set of questions was borrowed from the Creative Achievement Questionnaire^57^ and asked whether the subject had written a piece of original music, whether it had been performed, whether it had been published or recorded, and so on. The third set of questions asked whether the subject had composed or improvised original music, and, if so, how frequently, for how many years, and whether such activity was best described as improvising, writing songs, composing on paper, composing electronic music, or other. A final set of questions gauged general listening behaviors and preferences.

For this study, only the third set of questions was addressed, specifically the question as to how frequently subjects had improvised or written original music. Subjects responded on a scale from 1 to 6, with 1 representing never, and 6 representing several hours per day.

The relationship between “frequency” of creative acts (on the one hand) and “quality” of creativity (on the other) has long been discussed within the field. This is known as the “Equal Odds Rule” and is stated as follows: “The relationship between the number of hits (i.e., creative successes) and the total number of works produced in a given time period is positive, linear, stochastic, and stable”^58^. Relevant to the current study, this relationship has been shown to hold with classical composers^59^. While this relationship has previously been shown to hold in highly creative individuals (i.e., Big C), we recently demonstrated this relationship to exist, for the first time, in a cohort of normal, healthy, college students, most of whom overlap the current sample^52^. Thus, the frequency of participating in musical improvisation is an appropriate proxy measure for the resulting quality of such activities at a population level.

### Image Acquisition and Processing

Structural imaging was obtained at a 3 Tesla Siemens scanner using a 32-channel head coil to obtain a T1 5 echo sagittal MPRAGE sequence [TE = 1.64 ms; 3.5 ms; 5.36 ms; 7.22 ms; 9.08 ms; TR = 2530 ms; voxel size = 1.0 × 1.0 × 1.0 mm3; FOV = 256 mm; slices = 192; acquisition time = 6:03]. For all scans, each T1 was reviewed for image quality. Cortical reconstruction and volumetric segmentation were performed with the FreeSurfer-v5.3.0 image analysis suite, which is documented and freely available for download online (http://surfer.nmr.mgh.harvard.edu/). The methodology for FreeSurfer is described in full in several papers, and summarized by Reuter^60^. Briefly, this process includes motion correction and averaging of volumetric T1 weighted images, removal of non-brain tissue, automated Talairach transformation, segmentation of the subcortical white matter and deep gray matter volumetric structures, intensity normalization, tessellation of the gray matter, white matter boundary identification, automated topology correction, and surface deformation following intensity gradients to optimally place the gray/white and gray/cerebrospinal fluid borders. Segmented data were then parceled into units based on gyral and sulcal structure, resulting in values for cortical thickness, surface area, and volume. The results of the automatic segmentations were quality-controlled, and any errors were manually corrected. FreeSurfer morphometric procedures have been demonstrated to show good test-retest reliability across scanner manufacturers and across field strengths^60^.

### Statistical Analysis

A general linear model was used to assess correlations with musical creativity scale scores and cortical pial surface area. This type of group analysis was done by the Query, Design, Estimate, Contrast (QDEC) interface within FreeSurfer. QDEC is a single-binary application used to perform group averaging and inference on the cortical morphometric data created by the FreeSurfer processing stream (http://surfer.nmr.mgh.harvard.edu/fswiki/Qdec). First, the subject’s surface was smoothed using a full-width/half-maximum Gaussian kernel of 10 mm. This smoothing was done so that all subjects in this study could be displayed on a common template, which is an average brain. The design matrix consisted of musical creativity measures as the independent variable and age and sex as covariates, and the slope used was different offset/intercept, different slope (DODS). Correction for multiple comparisons was performed using a Monte Carlo Null-Z simulation method for cortical surface analysis available within QDEC. For these analyses, a total of 10,000 simulations were performed for each comparison, using a threshold of P = 0.05. This is the probability of forming a maximum cluster of that size or larger during the simulation under the null hypothesis and presents the likelihood that the cluster of vertices would have arisen by chance.

## Additional Information

**How to cite this article**: Bashwiner, D. M. *et al.* Musical Creativity “Revealed” in Brain Structure: Interplay between Motor, Default Mode, and Limbic Networks. *Sci. Rep.* **6**, 20482; doi: 10.1038/srep20482 (2016).

## Supplementary Material

Supplementary Information

## Figures and Tables

**Figure 2:**
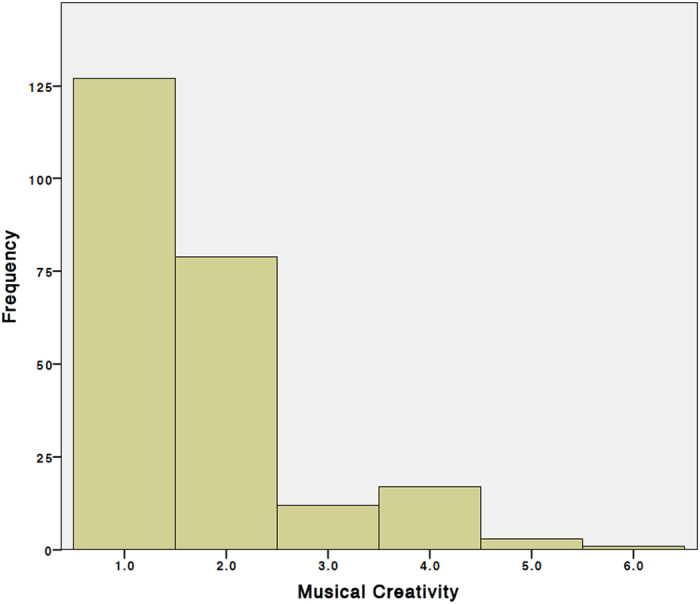
Distribution of Musical Creativity Scores across the entire sample (N=239). Question: “Have you ever improvised or written original music?” 1=Never, 2=Rarely, 3=Monthly, 4=Weekly, 5=Daily, 6=Several Hours/Day.

**Table 1:** Responses to Musical Creativity Questionnaire.

(*N* = 239)	*Males* (*N* = 123)	*Females* (*N* = 116)	(*t*)	*p*
Age	22.05 (3.6)	21.79 (3.5)	0.5	ns
Background^1^	3.46 (1.5)	3.78 (1.6)	1.5	0.12
Achievement^2^	2.67 (4.2)	2.30 (5.1)	0.6	ns
Creativity^3^	1.84 (1.0)	1.59 (0.9)	1.9	0.05*
General^4^	2.83 (1.5)	2.51 (1.3)	1.8	0.08
General^5^	5.33 (0.73)	5.47 (0.62)	−1.6	0.11

^1^Have you ever practiced a musical instrument?

^2^Musical Creative Achievement.

^3^Have you ever improvised or written original music?

^4^How musically creative would you rate yourself to be?

^5^How frequently do you listen to music? Values for males and females represent Mean and Standard Deviation (in parentheses). (t) = Student’s t statistic; p = significance level.

**Table 2:** Partial correlations, controlling for age and sex, between behavioral assessment measures commonly associated with creativity and subject scores on MCQ question III (“Have you ever improvised or written original music?”).

(*N* = 182)	*Musical Creativity*	*CAQ - Total*	*CAQ - Music*	*DT Originality*
CAQ - Total	0.31***			
CAQ - Music	0.56***	0.44***		
DT Originality	0.17*	0.23**	0.15*	
Openness/Intellect	0.20**	0.16*	0.20**	0.26***

MCQ—Musical Creativity Questionnaire; CAQ—Creative Achievement Questionnaire; DT—Divergent Thinking (Torrance Test of Creative Thinking); Openness/Intellect—Big Five Aspect Scale. *p < 0.05; **p < 0.01; ***p < 0.001.

**Table 3:** Musical Creativity Questionnaire: Regions surviving Monte Carlo simulation (P < 0.05).

Hem	Max	Size(mm^2^)	TalX	TalY	TalZ	P-Value	Vtxs	Gyrus
Left	4.915	3495.73	−10.4	−0.3	58.7	0.00010	6750	superior frontal
	4.114	1043.91	−34.6	−26.1	21	0.03840	2590	planum temporale
	3.617	2320	−26	16.7	−9.5	0.00010	4321	orbitofrontal
Right	5.883	2883.28	8.3	18.4	51.6	0.00010	5440	superior frontal
	4.567	1487.19	61.8	−15.6	−16.2	0.00510	2448	middle temporal
	2.912	1218.92	18.7	32.8	−17.4	0.01900	2008	lateral orbitofrontal
