# Supplementary material for: Musical Creativity “Revealed” in Brain Structure: Interplay between Motor, Default Mode, and Limbic Networks
Source: Sci Rep. 2016 Feb 18;6:20482. doi: 10.1038/srep20482 (PMC4757893; doi:10.1038/srep20482)
Supplement: Supplementary Information [file srep20482-s1.pdf]

Musical Creativity "Revealed" in Brain Structure: Interplay Between Motor, Default Mode, and Limbic Networks

David M. Bashwiner
Christopher J. Wertz
Ranee A. Flores
Rex E. Jung

Supplementary Materials

SUBJECT ID: ______________

MUSICAL CREATIVITY QUESTIONNAIRE

I. Musical Background

Have you ever practiced a musical instrument? Circle one option. (Please include "voice" as an instrument if you are a singer.)

1	2	3	4	5	6
Never	*Rarely*	*Monthly*	*Weekly*	*Daily*	*Several Hours/Day*

If 5 or 6, please indicate for each instrument:

a. The instrument played;
b. The number of years you played the instrument on a daily or almost daily basis;
c. The number of hours you practiced/played the instrument per day *averaged over the whole duration of the period indicated in (b);*
d. Whether study was predominantly formal (with a teacher), informal (for example, learning from recordings, self study from books, playing in a rock band), or an equal amount of both;
e. Whether such study predominantly involved reading music, playing by ear, or an equal amount of both.

**(a)** Instrument	**(b)** Years Played (Daily Basis)	**(c)** Average Number Hours Played	**(d)** Formality of Study (check one box only)			**(e)** Reading Music vs. Playing by Ear (check one box only)		
			Formal	Informal	Equal Amt. Both	Reading Music	Playing by Ear	Equal Amt. Both
1.								
2.								
3.								
4.								
5.								
6.								
7.								

II. Creative Musical Achievement

Place a check mark beside sentences that apply to you. For "recordings," consider both professional and semi-professional.

_____ I have composed an original piece of music.
_____ An original piece of music that I have composed has been published or recorded.
_____ I have performed on a recording of someone else's composition.
_____ I have performed on a recording of my own composition.
_____ Recordings of my composition have been sold publicly.
_____ My musical talent has been critiqued in a local publication.
_____ My compositions have been critiqued in a national publication.

III. Musical Creativity

Have you ever improvised or written original music? Circle one option.

1	*2*	*3*	*4*	*5*	*6*
Never	*Rarely*	*Monthly*	*Weekly*	*Daily*	*Several Hours/Day*

Please indicate for each behavior:

a. The number of years you have spent doing the specified behavior (enter 0 if none);
b. Whether your engagement with the behavior was daily, weekly, or infrequent.

	(a) Number of Years	(b) Degree of Engagement		
		Daily	Weekly	Infrequent
**Improvising**				
**Writing songs** (specifically the *musical* portions of songs—not lyrics				
**Composing on paper** (or in a notation program, such as Finale or Sibelius)				
**Composing electronic music**				
**Other (specify):** ______________________				

IV. General Questions

a. On a scale of 1 to 6 (6 = very creative), how musically creative would you rate yourself to be? (circle one number)

1	*2*	*3*	*4*	*5*	*6*
not creative					*very creative*

b. How frequently do you listen to music? (circle one)

1	*2*	*3*	*4*	*5*	*6*
Never	*Rarely*	*Monthly*	*Weekly*	*Daily*	*Several Hours/Day*

c. What type of music do you listen to most frequently? (circle one)

rock/pop jazz classical electronic folk rap other: __________
